# Applicability of Oral Fluid and Dried Blood Spot for Hepatitis B Virus Diagnosis

**DOI:** 10.1155/2019/5672795

**Published:** 2019-04-01

**Authors:** Livia Melo Villar, Cristianne Sousa Bezerra, Helena Medina Cruz, Moyra Machado Portilho, Geane Lopes Flores

**Affiliations:** ^1^Laboratory of Viral Hepatitis, Oswaldo Cruz Institute, FIOCRUZ, Rio de Janeiro, Brazil; ^2^Federal Institute of Education, Science and Technology of Ceará, Campus Fortaleza, Ceará, Brazil; ^3^Instituto Gonçalo Moniz, Fundação Oswaldo Cruz, Salvador, Bahia, Brazil

## Abstract

Hepatitis B virus (HBV) is one of the major causes of chronic liver disease worldwide; however most of individuals are not aware about the infection. Oral fluid and dried blood spot (DBS) samples may be an alternative to serum to HBV diagnosis to increase the access to diagnosis in remote areas or high-risk groups. The main objective of this review is to give an insight about the usefulness of oral fluid and DBS for detecting HBV markers. Several groups have evaluated the detection of HBsAg, anti-HBc, and anti-HBs markers in oral fluid and DBS samples demonstrating 13 to 100% of sensitivity and specificity according different groups, sample collectors, and diagnosis assays. In the same way, HBV DNA detection using oral fluid and DBS samples demonstrate different values of sensitivity according type of collection, studied group, extraction, and detection methods. Thus, serological and molecular diagnostic tests demonstrated good performance for detecting HBV using oral fluid and DBS according some characteristics and could be useful to increase the access to the diagnosis of HBV.

## 1. Introduction

Hepatitis B virus (HBV) infection is responsible for acute and chronic cases all over the world. Diagnosis of infection is made by detection of serological (HBsAg, HBeAg, anti-HBe, anti-HBc, anti-HBc IgM, and anti-HBs) and molecular markers (HBV DNA) [1, 2].

Enzyme immunoassay (EIA), electrochemiluminescence (ECLIA), chemiluminescence immunoassay (CLIA), microparticle enzyme immunoassay (MEIA), radioimmunoassay (RIA), and rapid assays have been used for serological diagnosis [1, 3, 4].

Molecular methods for detection and quantification of HBV DNA are important for (1) diagnosis of hepatitis B infection; (2) evaluation of disease prognostic evaluation the risk to cirrhosis and cancer risk; (3) defining the beginning of antiviral treatment; (4) monitoring antiviral treatment and identifying resistance to nucleus (t)ide analogues [1, 5, 6].

For the diagnosis of hepatitis B, serological and molecular detection are commonly performed using serum or plasma samples, which can be difficult in remote areas with scarce resources and potentially painful among individuals like drug users and patients under hemodialysis, obese, and elder people. However, alternative body fluids such as oral fluid and dried blood spots (DBS) have been studied as alternative fluids to serum for epidemiological and molecular diagnosis of HBV. Therefore, the objective of this review is to give new insight about serological and molecular diagnosis of HBV using oral fluid and DBS as alternative biological samples.

## 2. Oral Fluid Samples for Infectious Disease Diagnosis

Saliva is a body fluid comprising secretions from glands fed by the vasculature of the body, has an important role in maintaining oral health, with antiviral and antibacterial activity, in the lubrication and repair of oral mucosa, in the palate, and in a digestion, and is slightly acidic and composed of small organic substances, proteins, peptides, and polynucleotides [47]. Thus, many circulatory molecules (DNA, RNA, and proteins) are present in this fluid [48].

There are two important aspects that should be considered to collect saliva: the type (total or gland-specific) and the level of stimulation (stimulated or unstimulated) [49]. Total saliva originates mainly from salivary glands, the parotid, sublingual, and submandibular, and also contains gingival crevicular fluid (GCF); it is a plasma transudate, which constantly flows from the crevice between the gum margin and the teeth [50]; its collection is simple and does not require much equipment. Oral fluid contains principally GCF that is an ultrafiltrate of plasma that enters the oral cavity by transudation from capillaries present in the mucosa of the gingival space [13]; its collection coming from a specific gland and is a little more difficult [51, 52].

The diagnosis of some diseases using saliva or oral fluid samples is simple and noninvasive, being safe for both the professional and the patient. These characteristics make the collection of this fluid very attractive in children, the elderly and other individuals with difficult venous access [52]. On the other hand, some difficulties are observed when working with saliva due to their low IgG concentration compared to those observed in serum samples [50, 53] and the presence of enzymes with DNAs and RNAs activity that can degrade the DNA molecules, as well as inhibit their PCR amplification [48].

### 2.1. Usefulness of Oral Fluid to Detect HBV Antigen and Antibodies

The first reports for detecting HBV markers in saliva or oral fluids are from the decade 70's [54, 55]. Since then, several studies evaluated these samples as alternative to serum, but a large variation in the values of sensitivities and specificity was obtained. These differences could be the result of different oral fluid collector device, types of enzyme immunoassays (EIA), characteristics of populations, countries, and sample recruitment and size included. Some studies that compared saliva or oral fluid with serum to detect HBV markers are compiled in Table 1.

Some studies evaluated the detection of HBsAg in saliva and found that saliva could be used as an alternative sample for serum to identify HBV carriers [7, 8, 18, 55–60]. The oldest reports employed RIA as methodology assay for detecting HBV markers in saliva demonstrating sensitivity varying from 75% to 100% [54–57]. Most of studies detected HBsAg in saliva and oral fluid collected by different devices and different commercial EIAs with or without modifications on protocol [7, 8, 11, 13–18, 61–63]. Overall, these studies showed great sensitivity and specificity indicating the use of saliva or oral fluid samples at least for epidemiological studies to detect HBsAg.

Spitting samples were evaluated for the presence of HBsAg in acute HBV patients in Russia. Authors showed correlation between HBsAg detection in spitting and serum samples, but HBsAg were not detected earlier in saliva than serum in serial samples. They also observed high frequency of HBeAg in saliva compared to serum suggesting that saliva could be a source of HBV diagnosis and treatment monitoring [18]. Spitting samples were also evaluated for the presence of HBsAg compared with serum using a commercial EIA in other studies with values of sensitivity vary from 93.6% to 100% and specificity vary from 92.6% to 100% [8, 13, 15]. No modifications in EIA protocol were done in first study, while Cruz et al. [8] performed longer period of sample incubation period and modification of cut-off calculation and Khadseet al used longer period of sample incubation period [15].

Nowadays some oral fluid collection devices are commercialized, such as: Oracol (Malvern Medical Developments), Chembio (Chembio Diagnostic Systems, New York, USA), OraSure (OraSure Technologies, USA) and Salivette (Sarstedt, Germany). Some studies used these collectors to obtain oral fluid and compared to serum results for detecting HBsAg marker where values of sensitivity varied from 62% to 100% and specificity varied from 78.0% to 100% [7, 11, 13, 14, 16, 17, 61, 62].

High values of sensitivity and specificity were found for detecting HBV markers using Orasure, Oracol and Chembio collectors. HBsAg detection using oral fluid obtained by Orasure Collector and EIA with adaptations (increase of sample volume) demonstrated sensitivity and specificity of 100% [7, 61]. Oracol collector and EIA with long period of sample incubation demonstrated values of HBsAg sensitivity and specificity of 90.7% and 100%, respectively [11]. Chembio collector and EIA without modifications for HBsAg detection showed sensitivity and specificity of 95.2% and 100%, respectively [14].

Studies evaluating Salivette device observed lower sensitivity and specificity values compared to other devices for detecting HBV markers. Salivette and EIA without modifications demonstrated sensitivity from 78% to 92.0% and specificities from 86.8% to 89.9% [14, 62]. On the other hand, EIA with modifications (extension of sample incubation period and ROC curve analysis for cut-off calculation) along to Salivette device allowed HBsAg sensitivity of 85.1% among monoinfected HBV patients [13] and 80.9% between HBV/HIV coinfected individuals [17]. These results demonstrated the importance of modifications in EIA protocol for testing oral fluid and the influence of HIV status in the performance of assay.

Anti-HBc IgM to identify acute HBV was also tested in samples obtained by spitting and Salivette device where detectable anti-HBc IgM were found in oral fluid, but the number investigated (8) was insufficient to draw firm conclusions on the usefulness of salivary IgM anti-HBc detection for diagnosing recent HBV infection [64]. Orasure Collector and EIA with increase in oral fluid volume provide sensitivity and specificity of 100% [7, 12]. Flores et al. also investigated the usefulness of Salivette device along to EIA for detecting anti-HBc and found sensitivity of 82.4% and specificity of 96.9% demonstrating the presence of this marker in oral fluid. Amado et al. [12] also evaluated anti-HBs marker in Orasure demonstrating high specificity (100%) and low sensitivity (8.3%), but few samples were included in the study

### 2.2. HBV DNA Detection in Oral Fluid Samples

The studies concerning HBV DNA detection in oral fluid samples started in the years 2000. A study conducted by Noppornpanth and colleagues [8] evaluated spitting samples for HBV DNA detection through an in-house qualitative PCR and found a sensitivity of 47.8%. Furthermore, they demonstrated that HBV DNA detection in saliva samples is higher when patients have detectable HBsAg and HBeAg. In the same year, a study conducted among military men serving in the West Siberian region showed a similar sensitivity of HBV DNA detection (46.2%) among oral fluid samples using the same collection method and qualitative in-house PCR during the acute period. On the other hand, during early convalescence, HBV DNA was found in the blood of only one patient and was not found in saliva.

Some authors evaluated detection of HBV DNA using in-house real-time PCR methodologies and spitting samples. In the study of Van der Eijk et al. [19], 47% of patients with serum positivity of HBV DNA also had detectable HBV DNA in saliva using a TaqMan protocol and an automated extraction protocol of viral DNA (Magnapure LC isolation station, Roche Applied Science, Penzberg, Germany) with a modified protocol that included initial proteinase K digestion. Furthermore, they found HBV DNA in saliva of most of HBeAg positive individuals (76%). On the other hand, low sensitivity of HBV DNA in saliva (21.7%) was found probably due to the different DNA extraction method used (phenol-chloroform extraction) [20]. Zhang et al. [21] evaluated this type of samples from HBV chronic carriers using in-house real-time PCR and found sensitivity of 72.5% in saliva compared to paired serum. Furthermore, these authors demonstrated that when the serum contains high HBV DNA viral load, the content of saliva HBV DNA virus should be likely high (mean serum viral load of 6.63 ± 1.55 log copies/ml and corresponding mean saliva viral load of 5.21 ± 1.85 log copies/ml).

More recently, spitting samples from 50 chronic carriers were submitted to a commercial method for HBV amplification and quantification (Roboscreen GmbH/Analytik Jena Group) followed by DNA extraction with QIAamp DNA Kit (Qiagen; Venlo, Limburg, The Netherlands). In this study, the sensitivity of HBV DNA detection in saliva was 68% and mean viral load among oral fluid samples was 3.8x10^4^ ± 5.42x10^4^ copies/mL when viral load is > 10^7^ in paired serum samples [23].

Some studies suggest that collectors of oral fluid that uses mechanical friction to the mouth mucosa present higher efficiency. In 2010, Heiberg and colleagues [22] demonstrated sensitivity of 92% for detecting HBV DNA in oral fluid samples from children with chronic hepatitis B. These samples were collected with Oracol (Malvern Medical Developments, Worcester, United Kingdom) that must be rubbed through the gum and then proceed to HBV DNA purification with MagNa Pure LC Instrument (Roche Applied Science, Penzberg, Germany) and an in-house real-time PCR. In 2012, Portilho et al. [65] evaluated two oral fluid collectors, Salivette and Chembio and three in-house qualitative PCR protocols for detecting HBV DNA and found that Salivette along to PCR for Core gene presented the most satisfactory results (sensitivity of 20 copies of HBV DNA/mL) showing that mechanical friction not always generate the best results. Furthermore, the same authors compared four collection methods (Salivette, spitting, DNA-Sal and FTA Cards) for HBV DNA detection using a qualitative in-house PCR for polymerase gene among samples from chronic carriers of HBV [24]. In this study, best results were obtained using Salivette device, in particular, among individuals presenting HBeAg and high HBV DNA viral load in serum. Using quantitative PCR and Salivette device, the results were not satisfactory, since sensitivity was 18.2% [25].

Oral fluid samples collected with Salivette were also evaluated for detection of HBV DNA among patients with occult hepatitis B infection (OBI) [26]. In this study, it was possible to detect HBV in 4 saliva from 5 OBI cases and it was not detected in the individual that had low viral load (1.623 log IU / mL) in serum.

Information regarding these studies are compiled and described in Table 2.

## 3. Usefulness of DBS to HBV Diagnosis

Some studies have reported the use of DBS for diagnostic screening tests for human immunodeficiency markers of virus (HIV) and hepatitis virus [30, 66–69]. The use of alternative clinical specimens such as saliva and DBS may facilitate access to diagnosis, since collection method is simpler and does not require a highly specialized professional. In addition, transport and storage of samples can be performed at room temperature, which facilitates collection in distant areas of the laboratory. This type of sample also facilitates the collection of blood from children and individuals with difficult venous access [34, 39, 40].

Despite the numerous benefits, it is worth to mention that the use of this type of material present some difficulties. The small amount of sample disposed in the filter paper cards may limit the number of analyzes to be performed on that material if the collection has not been satisfactorily performed. The DBS drying process may interfere with subsequent analyzes of the sample, since dried blood can carry disrupted cells and a large amount of cellular constituents and may modify the biochemical structure of the molecule to be quantified (antibodies, for example). In addition, it is not easy to adapt commercial tests designed to serum or plasma analysis to be used on DBS samples. In addition, the concentration of the substances in serum and whole blood differ, which may raise the need to propose a correction factor that approximates the quantitative results obtained in the DBS from the serum values of paired samples in cases where the serum analysis is the “gold standard” diagnosis [70].

It is understood that the use of DBS in the diagnosis of infectious diseases is possible but must undergo rigorous studies of validation and evaluation of sensitivity and specificity that also involve a good knowledge of the target microorganism [71, 72].

Diagnosis of HBV is usually performed on blood samples obtained by venous puncture. However, obtaining this sample is difficult in some cases, as in regions where there is no access to an appropriate laboratory. In addition, the transport of serum or plasma samples from remote areas to central laboratories is also complicated. Because of this, it is important to use alternative biological samples capable of providing an adequate diagnosis.

### 3.1. HBV Antibody and Antigen Detection Using DBS Samples

Initial studies using DBS to detect HBsAg, HBeAg, and anti-HBs occurred in the 1970s. In this period, the positive aspects of the use of this strategy, including results comparable to those obtained from serum samples, were already emphasized [73–76]. In the 1990s, Gupta innovated the use of DBS, conjugating it to the detection of HBV by PCR and finding a detection limit of 10^2^ viral particles [38]. Studies of large populations, as well as research evaluating sensitivity, specificity and stability in different storage temperatures emphasize the applicability of this method [27, 30, 36, 77].

Detection of HBV antibodies and antigen, such as HBsAg, anti-HBc and anti-HBs by enzyme immunoassays (EIAs) can also be obtained through DBS samples [30]. The HBsAg marker was the first marker to be investigated, it is found around 4 weeks after infection, and important indicator of active infection [1]. Some studies are demonstrated in Table 3.

The studies for detecting HBV in DBS evaluated several factors, such as: size of disc paper used, ranging from 6mm-12mm, sample amount (50*μ*l-100*μ*l), cut-off value (0.100nm-1.99nm), and storage temperature (room temperature to -80°C) in different commercial EIA kits.

A factor highlighted in several studies was the cut-off value for determining the positive and negative samples; this value could be higher for DBS samples than for plasma or serum samples. The volume of DBS eluate was also considered, mostly between 50 *μ*l and 100 *μ*l. Low sensitivity (79%) and specificity (89%) [29] were reported when 25 *μ*l of DBS was used in detection of HBsAg [78].

Several studies have demonstrated agreement in the detection of HBV markers between DBS samples and serum samples in EIAs. Cruz* et al.* [79] demonstrated sensitivity and specificity of 100% using Imunoscreen HBsAg SS, a commercial assay for detecting HBsAg in DBS.

In a meta-analysis, 23 studies were included and assays such as Enzygnost (Behring) and ARCHITECT (Abbott) showed HBsAg sensitivity and specificity of 98% (95% CI: 95%-99%) and 100% (95% CI: 99-100%), respectively. Other studies demonstrated sensitivity and specificity values to detect HBsAg in DBS from 78.6 to 98.6% and 88.6 to 100.0%, respectively [29–32, 35, 37].

In the evaluation of HBsAg in DBS in the presence of HIV coinfection, Flores et al. [17] showed that it is possible to detect HBsAg and obtained 90% of sensitivity among monoinfected HBV+ individuals and 80.9% in HIV/HBV+ patients using commercial EIA. Mossner et al. [46] stated that the sensitivity of HBsAg detection in HIV / HBV + individuals is likely to be lower due to treatment with antiretrovirals. Indeed, Flores et al. [17] observed that most discordant results occurred at HBV/HIV+ patients who were under antiretroviral treatment.

Anti-HBc marker that indicates previous exposure to HBV was also evaluated under various parameters such as disc sample size, sample eluate, cut-off value, and storage temperature [30] demonstrating the importance of cut-off value to adapt commercial EIA for detecting anti-HBc in DBS. McAllister* et al.* [37] evaluated storage temperature of DBS and showed that -20°C or -70°C are the ideal temperatures to storage and that freezing should occur as soon as possible after collection.

Other studies also evaluated the detection of anti-HBc in DBS and presented sensitivities and specificities ranging from 90% to 97% and 92.6% to 100%, respectively [30, 31, 37] showing the applicability of DBS for detecting anti-HBc using different assays.

A recent study evaluated the prevalence of anti-HBc in prisoners in Scotland using DBS along to Architect Hepatitis B core II antibody test [80]. This study shows that the use of DBS in diagnosis is already being used for epidemiological screening. Flores et al. [17] demonstrated similar sensitivity for detecting anti-HBc in DBS from monoinfected HBV+ group (77%) and in HIV/HBV^+^ coinfected (76%).

Some studies also evaluated anti-HBs detection in DBS samples and found sensitivity and specificity of 74.2 to 97.5% and 86.9 to 100%, respectively [30–32, 35]. Some methods seem more effective for detecting anti-HBs in DBS, such as chemiluminescent microparticle immunoassay that gave sensitivity and specificity above 97% for detecting anti-HBs in DBS [31]. On the other hand, EIA test found 76% of sensitivity in the detection anti-HBs even using the larger disc of paper (12.5mm) than those used for detecting HBsAg and anti-HBc (6 mm) [30]. A correlation between HBV results in paired DBS and serum samples showed that DBS could be an alternative biological specimen to replace serum for HBV diagnosis, but adaptation of sample processing and detection method is critical.

Lee et al. [35] reported the importance of blood volume used in the filter paper and the sample dilution volume. They applied 50 *μ*L of blood in paper and sample was eluted in 500 *μ*L of diluent, thus further diluting the sample what could result in low sensitivity (74.9%). Brown* et al*. [28] demonstrated high sensitivity in the qualitative test, but low anti-HBs titers were observed in DBS compared to paired serum. On the other hand, Flores et al. [81] found that DBS can be used to detect and quantify anti-HBs what could increase the access to identify susceptible people.

### 3.2. Molecular Diagnosis of HBV Using DBS Samples

As previously mentioned, Gupta innovated HBV diagnosis when proposed the role of DBS for detecting HBV DNA [38]. This study was conducted in India with HBsAg reactive serum samples mixed with HBsAg negative whole blood obtained from donors. After the mixture, samples were spotted on 7-mm disks of Whatman 3MM filter paper or a nitrocellulose membrane (BA85; Schleicher & Schuell). Disks were punched and analyzed by a simple PCR protocol targeting the core gene of HBV. PCR results were acquired after gel electrophoresis and staining with ethidium bromide. Gupta and his colleagues estimated HBV detection limit of 10^4^ virus particles when PCR was realized without hybridization methods [38]. According to the authors, the sensitivity of the test could be improved with the use of hybridization method after gel electrophoresis or with the increase of the number of cycles of the PCR. They did not determine the sensitivity and specificity of the test, because they did not analyze a group of positive and negative individuals.

In 2004, Jardi and colleagues [39] proposed an advance in the use of the DBS for HBV molecular diagnosis. While Gupta et al. [38] used DBS to detect HBV DNA, Jardi et al. [39] applied this type of sample to quantify and genotype HBV DNA. In this way, they evaluated paired samples (serum and DBS) obtained from 82 HBsAg-positive patients. For both samples, serum and DBS, viral DNA was extracted with QIAamp minikit (Qiagen Ltd., Sussex, UK) and HBV DNA quantification was performed with two sets of PCR primers and probes targeting HBV core gene. In addition, HBV genotypes were determined by restriction fragment length polymorphism of the S gene sequence amplified by PCR. After the analyses, the authors concluded that DBS could be used to quantify HBV DNA and to characterize HBV genotypes. However, the agreement of serum and DBS quantitative results were better in samples showing HBV DNA serum levels above 1 x 10^3^copies/mL.

In 2009, Lira and colleagues evaluated the utility of DBS samples for monitoring HBV infection in patients from Mexico City [40]. They analyzed 47 HBsAg-positive individuals that gave paired plasma and DBS and used QIamp® DNA micro kit (QIAGEN GMBH, Germany) as extraction method and commercial method to quantify HBV (HBV Monitor Cobas Amplicor v1.5, Roche Diagnostics, New Jersey, EUA). They found an excellent sensitivity value of the 100% and conclude that HBV DNA concentration from DBS is highly comparable to plasma paired values. Moreover, Lira and colleagues [40] did not find a significant difference in DNA detection when DBS was stored at -20°C, 4°C, 20°C or 37°C for 7 days.

After 2009, several studies evaluated DBS to detect and quantify HBV DNA [31, 32, 41–46, 78]. In 2013, a study investigated the performance of DBS in HBV diagnosis using commercial tests in comparison to results obtained in plasma samples [32]. They used FTA DMPK-C card (Whatman, GE Healthcare, NJ, EUA), indicated for molecular analyses along to commercial real-time PCR to HBV DNA quantification (CobasTaqman HBV test v2.0), and found limit of HBV DNA detection in DBS of 914.10 ± 157.77 IU/mL. They showed a good correlation between DBS and plasma HBV viral load and sensitivity of 98%.

Two years later, in 2015, a research group evaluated the application of DBS to HBV DNA quantification in coinfected samples (HBV+ and HIV+) [44]. For this, they used Whatman 903 protein saver DBS cards (GE Healthcare, New Jersey, USA) and DNA extraction with a preextraction (SPEX) buffer (Roche Molecular Systems, Pleasanton, California, USA), associated with stage of incubation at 56°C for 10 minutes and AmpliPrep/COBAS TaqMan HBV test (Roche, v2.0, California, USA) as detection method. They observed that sensitivity of HBV DNA detection in DBS increased according serum HBV viral load, since the probability of undetectable HBV in DBS was higher when plasma had viral load of 200 IU/ml compared to DBS samples whose paired serum had viral titers above 2000 IU/ml.

In 2016, in Ethiopia, Stene-Johansen and colleagues [45] used DBS for HBV monitoring under real-life conditions in Africa. To do this, they collected plasma and whole blood from hepatitis B patients that presented plasma viral load ranging from 2.14 log to >7 log IU/ml. The whole blood was applied at Whatman 903 sample collection cards (GE Healthcare Life Sciences, Norway). Abbott RealTime HBV assay on sp2000 extractor (Abbott Molecular, DesMoines, IL, USA) was used to extract the HBV DNA and the rt2000 real-time PCR instrument (Abbott Molecular, DesMoines, IL, USA) was used to perform HBV DNA quantification in DBS and plasma. Stene-Johansen and colleagues [45] calculated a dilution factor based on the volume of whole blood used in quantitative assay to correlate plasma and DBS viral load and found good concordance between HBV viral load in plasma and DBS.

In 2016, Mossner and colleagues [46] determined and compared the sensitivity of HBV DNA detection in DBS compared to whole plasma. In contrast to other studies, they obtained whole blood from the fingertip(s) of the participants and not from venipuncture. The whole blood was distributed on a Whatman® 903 protein saver card (Sigma-Aldrich, Copenhagen, Denmark) and HBV DNA detection was made using Ultrio Elite assay, a type of qualitative nucleic acid amplification test (NAT) for the detection of HIV-1/2 RNA, HCV RNA and HBV DNA that demonstrated sensitivity of 97.6%. Main information regarding studies that used DBS for HBV DNA detection is compiled and described in Table 4.

## 4. Conclusion

HBV antigen, antibodies, and DNA could be detected in DBS and oral fluid samples using different devices and detection methods. Different values of sensitivity and specificity were found for HBV detection in saliva or oral fluid samples according type of collection device, EIA, extraction, and PCR method. It is interesting to observe that HBeAg status and HBV DNA presence in serum are positively related to HBV DNA detection in oral fluid. As well as HIV status or antiretroviral treatment could interfere in the performance of HBsAg and anti-HBc detection in oral fluid.

With respect to DBS, HBV detection was made using different sizes of disc paper, elution volume, and cut-off values. For each marker, it is important to optimize the assay. Sometimes dilution factors were employed to approach quantitative results obtained in DBS from that one acquired at the standard samples. For molecular HBV detection in DBS, high sensitivity and more accurate results were found using commercial methods. However, commercial methods are expensive which can hamper the employment of these tests in low resource areas. Despite this, studies show that HBV DNA could be detected using molecular methods.

## Figures and Tables

**Table 1:** Main Characteristics of studies that detect HBV antigen and antibodies in saliva or oral fluid samples.

Marker	Population	Country	Oral fluid device or sample collection	Assay	Sensitivity	Specificity	Reference

HBsAg	Local and out-of-state public health clinics	USA	Orasure	Commercial Enzyme immunoassay	100.0%	100.0%	[7]
Anti-HBc IgM	Local and out-of-state public health clinics	USA	Orasure	Commercial Enzyme immunoassay	100.0%	100.0%	[7]
HBsAg	Patients with and without HBV	Thailand	Whole saliva	Commercial Enzyme immunoassay	96.5%	100.0%	[8]
Anti-HBc	Rural community	Ethiopia	Sponge swab	Commercial Enzyme immunoassay	43.0%	87.0%	[9]
Anti-HBc	blood donors and injecting drug users	Denmark	Omni-Sal	Commercial Enzyme immunoassay	85.9%	100.0%	[10]
HBsAg	Unit of Gastroenterology of three university hospitals	Belgium	Oracol	Commercial Enzyme immunoassay	90.7%	100.0%	[11]
Anti-HBc Total	Viral Hepatitis ambulatory	Brazil	Orasure	Commercial Enzyme immunoassay	13%	100.0%	[12]
Anti-HBc IgM	Viral Hepatitis ambulatory	Brazil	Orasure	Commercial Enzyme immunoassay	100.0%	100.0%	[12]
Anti-HBs	Viral Hepatitis ambulatory	Brazil	Orasure	Commercial Enzyme immunoassay	8.33%	100.0%	[12]
HBsAg	Viral Hepatitis ambulatory	Brazil	Salivette	Commercial Enzyme immunoassay	85.1%	94.1%	[13]
			Whole saliva		93.6%	92.6%	
HBsAg	Viral Hepatitis ambulatory and Pantanal of Mato Grosso do Sul	Brazil	Chembio	Commercial Enzyme immunoassay	95.2%	100%	[14]
					78.3%	89.9%	
			Salivette				
HBsAg	Patients with and without HBV	India	Whole saliva	Commercial Enzyme immunoassay	100%	100%	[15]
HBsAg	Alcoholics	Brazil	Salivette	Commercial Enzyme immunoassay	Not available	100%	[16]
HBsAg	Viral Hepatitis ambulatory	Brazil	Salivette	Commercial Enzyme immunoassay	80.9%	86.8%	[17]
Anti-HBc					82.4%	96.9%	

**Table 2:** Main characteristics of studies that detect HBV-DNA in saliva or oral fluid samples.

Reference	Type of saliva or collection device	Population	Serum HBsAg or HBV	Molecular method	Sensitivity of HBV DNA
					Detection in oral fluid
			DNA positive subjects (n)		
[7]	Whole saliva	Thailand HBV carriers	23	In-house qualitative PCR	47.8%
[18]	Whole saliva	Siberian military men	42	In-house qualitative PCR	46.2%
[19]	Whole saliva	HBV chronic carriers	147	In-house real-time PCR	47%
[20]	Whole saliva	HBV chronic carriers	23	In-house real-time PCR	21.7%
[21]	Whole saliva	HBV chronic carriers	200	In-house real-time PCR	72.5%
[22]	Oracol	Children with chronic HBV	46	In-house real-time PCR	92%
[23]	Whole saliva	HBV chronic carriers	50	Commercial PCR	68%
[24]	Salivette, FTA Cards, DNA-Sal and whole saliva	HBV chronic carriers	32	In-house qualitative PCR	53.12%
[25]	Salivette	HBV chronic carriers	55	In-house real-time PCR	18.2%
[26]	Salivette	Occult HBV carriers	5	In-house qualitative PCR	80%

**Table 3:** Main characteristics of studies that detect HBsAg, anti-HBc, and HBsAg in DBS compared to serum.

Marker	Population	Country	Type of Assay	Specificity	Sensitivity	Reference
HBsAg	Pregnant women	Brazil	Enzyme Immunoassay	100%	100%	[27]
HBsAg	Unclear	UK	chemiluminescent microparticle immunoassay	100%	98%	[28]
HBsAg	Inpatients	Germany	Not specified	99.8%	91.7%	[29]
HBsAg	Patients attending HCV clinic	Brazil	Enzyme Immunoassay	97%	98%	[30]
HBsAg	Unclear	Germany	chemiluminescent microparticle immunoassay	100%	99%	[31]
HBsAg	Prospective patients from hepatitis clinic and blood donors	France	chemiluminescent microparticle immunoassay	98%	100%	[32]
HBsAg	Attendees of HIV testing center	Burkina-Faso	Rapid test, Enzyme Immunoassay, chemiluminescent microparticle immunoassay	100%	96%	[33]
HBsAg	Broad	Gambia	Rapid test	100%	96%	[34]
HBsAg	Patients at a tertiary hospital	Malaysia	chemiluminescent microparticle immunoassay	98%	97%	[35]
HBsAg	Unclear	US	chemiluminescent immunoassay	100%	100%	[36]
Anti-HBc	Unclear	UK	chemiluminescent microparticle immunoassay	100%	100%	[37]
Anti-HBc	Patients referred to the Viral Hepatitis Clinic	Brazil	Enzyme Immunoassay	92.6%	90.5%	[30]
Anti-HBc	Patients referred to the Viral Hepatitis Clinic	Brazil	Enzyme Immunoassay	NR	90.4%	[17]
Anti-HBc	Unclear	Germany	chemiluminescent microparticle immunoassay	100%	97%	[31]
Anti-HBs	Hospital	Malaysia	chemiluminescent microparticle immunoassay	86.9%	74,2%	[35]
Anti-HBs	Prospective patients from hepatitis clinic and blood donors	France	chemiluminescent microparticle immunoassay	100%	98%	[32]
Anti-HBs	Unclear	Germany	chemiluminescent microparticle immunoassay	100%	97.5%	[31]
Anti-HBs	Patients referred to the Viral Hepatitis Clinic	Brazil	Enzyme Immunoassay	97.3%	78%	[30]

**Table 4:** Main characteristics of studies of detection of HBV-DNA from DBS samples compared to serum or plasma.

Reference	Population	Country	DNA Extraction Method (DBS)	Amplification Method	Sensitivity (%)	Specificity (%)
[38]	HBsAg positive serum + negative whole blood	India	Not performed	Simple PCR (core gene)	NR	NR
[39]	HBsAg positive patients (82)	Spain	QIAamp mini columns	Real Time PCR (core gene)	88	100
[40]	HBsAg positive patients (47)	Mexico	QIAamp® DNA micro kit	Cobas Amplicorv1.5	100	NR
[41]	60 patients with undetermined serological Status	China	Chelex-100	Nested PCR (pre-core/core gene)	NR	NR
[42]	HBsAg positive patients (50)	Egypt	Not performed	Direct amplification (KAPA blood PCR Kit)	100	100
	HBsAg negative patients (10)					
[31]	HBV DNA positive patients (100)	Germany	MagNaPure 96 system using Viral NA Universal kit	Artus HBV LC PCR VERSANT HBV bDNA 3.0 assay	93	100
	HBV DNA negative patients (50)					
[32]	HBV DNA positive patients (50)	France	FTA purification reagent	Cobas Taqman HBV test V2.0	98	NR
	HBV DNA negative patients (10)					
[43]	HBsAg positive patients (26)	Congo	COBAS® AmpliPrep/COBAS® Taqman® HBV test v1.0	COBAS® AmpliPrep/COBAS® Taqman® HBV test v1.0	96	NR
[29]	HBV DNA positive patients (100)	Germany	Not informed	Not informed	93	NR
[44]	HBV DNA positive and HIV-infected patients (68)	Zambia	Pre-extraction buffer	AmpliPrep/COBAS TaqMan HBV test	91	NR
[45]	HBV viral load value range from 2.14 log to *>*7 log IU/ml	Ethiopia	Abbott RealTime HBV assay on sp2000 extractor	rt2000realtime PCR instrument	88	NR
[46]	Hepatitis B-infected patients (85)	Denmark	Not performed	Ultrio Elite assay	97.6	NR
	Blood donors negative for HBV infection (99)					

*NR*: not reported.
